# The effect of trans-provincial immediate reimbursement for healthcare expenses on inequity in the utilization of healthcare services for migrants

**DOI:** 10.1186/s12939-024-02096-5

**Published:** 2024-03-11

**Authors:** Mei Zhou, Longwei Zhang, Yunjia Liang, Yuxiao Chen

**Affiliations:** 1 School of Public Administration, Southwestern University of Finance and Economics, Chengdu, Sichuan China; 2 Department of Economics, Sichuan University, Chengdu, China; 3 School of Politics and Public Administration, Zhengzhou University, Zhengzhou, China

**Keywords:** Migrants, Immediate reimbursement, Health insurance, Healthcare services utilization

## Abstract

**Background:**

Improving the accessibility of public services for migrants is an important endeavor to promote equity in economic and social development. As a response to the large-scale movement of migrants and the fragmentation of China’s health insurance system, the Chinese Government has launched a policy of trans-provincial immediate reimbursement for healthcare expenses. The present study hopes to examine the effect of immediate reimbursement policy on the utilization of healthcare services for migrants in China.

**Methods:**

This study used two waves of data from the China Migrants Dynamic Survey (CMDS) collected in 2013 and 2017, with the sample comprising 13,540 individuals. We constructed a difference-in-differences (DID) model to investigate the impact of the policy on the utilization of healthcare services for migrants. Meanwhile, we also analyzed the heterogeneity of the policy effect by grouping the samples by industry, gender, income, and education level.

**Results:**

The results found that the trans-provincial immediate reimbursement significantly promoted the probability of migrants’ utilization of quality healthcare services (average treatment effect on the treated = 0.072, *p* < 0.05). Heterogeneity analyses revealed that the policy effect was more pronounced among higher-income and better-educated migrants. In addition, the policy effect was more significant for female migrants, and the benefits were more marked for migrants in high-risk industries.

**Conclusions:**

The trans-provincial immediate reimbursement policy has improved the inequity of healthcare services utilization among migrants as a whole; however, within the migrants, inequity still exists. More attention should also be paid to low-income or low-education groups in future policy design.

## Background

**Fig. 1:**
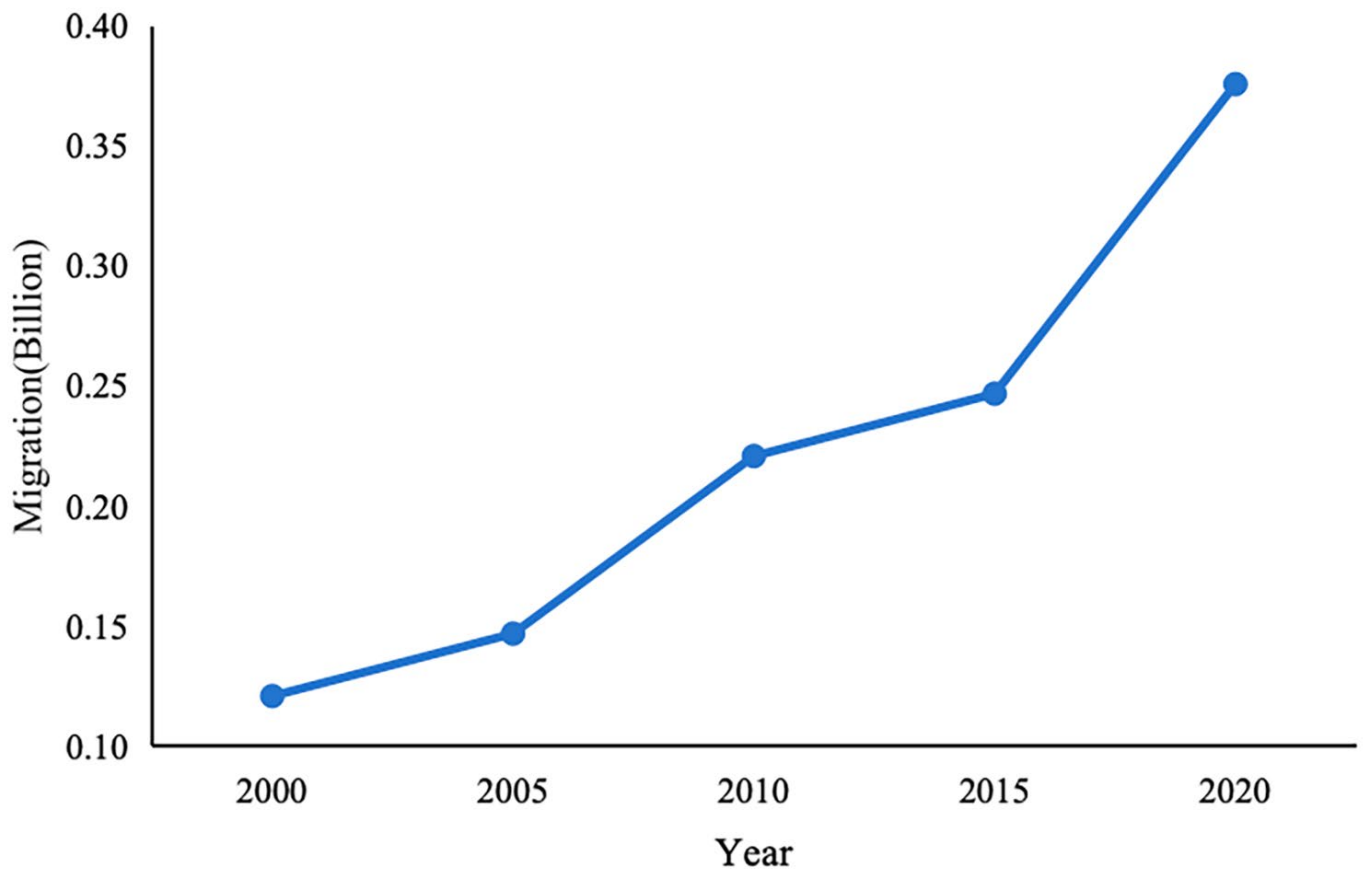
Trends in the scale of migration in China

The imbalance in economic development between regions has created differences in the demand for the labor force, which leads to large-scale and regular migration. The number of migrants in China is huge and has been increasing. According to the data from China’s seventh national census, the number of migrants in China has reached 376 million, showing an increase of 69.73% compared with the sixth national census in 2010^1^ (see Fig. 1), and is expected to remain high growth in the future [1]. There are obvious inequities between the migrants and the locals in terms of social welfare and social security benefits [2–5], thus deriving the problem of inequitable and inadequate access to basic public services for the migrants. This issue has gradually become a hot topic of concern for the Chinese government and academics [6–8], mainly in such areas as public education [9–11], labor and employment [12–13], and basic housing benefits [14–15]. The use of healthcare services for migrants is also an issue of greater concern to academics. Literature studies have found that most migrants do not have equitable access to healthcare services in their inflow cities, and there is a large gap between the demand and supply of healthcare services for migrants [16–17].

The fragmentation of China’s basic health insurance system is considered to be the main cause of inequitable access to healthcare services for migrants. Although China has achieved universal health insurance, the system uses the principle of “Localization management”. According to this principle, residents participate in the basic health insurance at their hukou registration place, which is divided into city-level administrative units, and the funds are also used and managed in each city. This is similar to the mechanism for cross-border medical treatment by citizens of European Union member states, with a distinction between “local” and “foreign”, and is divided into treating member state (EU member state where citizens receive the treatment) and the belonging member state (EU member state where citizens are insured or enjoy the right to disease allowance according to the laws of that member state), and also face problems such as reimbursement of cross-border medical expenses [18]. Since there is a policy threshold for migrants to enroll in health insurance in the inflow areas, most migrants are forced to enroll in health insurance in the place of household registration, and the fragmentation of the health insurance system has led to a distinction between “local residents” and “foreign residents” in accessing healthcare services. However, the health insurance funds are not interoperable between the inflow cities and the outflow cities, the advance payment of medical expenses and differences in regional reimbursement system increase the cost of medical treatment for migrants, and the accessibility of conventional healthcare services for migrants is low [19]. The liquidity constraints caused by declining reimbursement rates and advance payments significantly reduce the convenience of health insurance and suppress the basic healthcare needs of the migrants [20–21], resulting in a double loss of fairness and efficiency in health insurance. In addition, during the period from 2013 to 2018, the highest and lowest health record filing rates for the migrants were 38.44% and 22.99%^2^, respectively. This means that the majority of the migrants have not been included in the health record management in the inflow areas, and therefore cannot fairly use the healthcare services in the inflow cities. The coverage rate of health education, health management rate of children, and preventive inoculation rate of migrants in most areas are far lower than those of locals [22].

To solve the problem of inequity and inadequate utilization of healthcare services for migrants caused by the fragmentation of health insurance, the Chinese government decided to implement the policy of immediate reimbursement for trans-provincial healthcare services, which aims to ensure the basic rights of residents to trans-provincial medical treatment and improve the accessibility of residents to high-quality medical resources. By the end of 2022, the number of trans-provincial networked medical institutions nationwide was 62,700, with 5,687,900 visits for trans-provincial immediate reimbursement, and the fund paid out 76.233 billion yuan. The policy of trans-provincial immediate reimbursement of healthcare services expenses has been rapidly launched in China, which alleviates the pressure of advance payment and liquidity constraints for trans-provincial patients, especially migrants. On the one hand, immediate reimbursement allows migrants to access high-quality healthcare resources in the city of inflow at a lower cash expense. On the other hand, immediate reimbursement reduces the transaction costs for migrants who have to return to their hometowns for reimbursement afterward. The healthcare needs of migrants may be unleashed, and illnesses that would usually be foregone due to liquidity constraints may be treated in a more timely and regular manner, thus promoting the utilization of high-quality healthcare resources by migrants.

Taking the implementation of trans-provincial immediate reimbursement as the policy background, this paper studies the impact of immediate reimbursement on the utilization of healthcare services for migrants, using two waves of individual-level data from the China Migrants Dynamic Survey (CMDS) collected in 2013 and 2017. Based on the list of cities released by the Ministry of Human Resources and Social Security of the People’s Republic of China (MOHRSS) in real-time, this paper constructs a difference-in-differences (DID) model to identify the net effect of policy, and further discusses the heterogeneity of policy effects in different industry risks, income, education groups.

## Methods

### Data source

The data from the CMDS collected in 2013 and 2017 are used in this paper. The CMDS data are surveyed by the National Health Commission of the People’s Republic of China (NHC), using the annual population reports of 31 provinces as the basic sampling frame, and the target survey respondents are migrants aged 15 years or older who have resided in the inflow city for more than one month. CMDS data covers the basic characteristics of family demographics, employment and consumption, residence willingness and social integration, basic public health services and other modules. It is a large-scale micro monitoring database specifically designed for the survival status of migrants in China. There are great differences in the questionnaire settings of the healthcare services utilization in each survey year, and the variables between different years cannot achieve a good correspondence. This paper selects two waves of samples in 2013 and 2017, which not only meet the correspondence of the relevant variables of healthcare service utilization, but also cover the sample period before and after the implementation of the trans-provincial immediate reimbursement policy.

According to the study design, the data cleaning in this paper is as follows. First, this study aims to evaluate the impact of trans-provincial immediate reimbursement on the utilization of healthcare services for migrants, so only the trans-provincial migrants are retained in the data, and the intra-provincial migrants are excluded. Second, we drop migrants who repeat enrolment in health insurance, are not enrolled in health insurance, or are enrolled in health insurance in the inflow city. Third, the data of migrants in 2013 and 2017 are collated into panel data after removing the sample of missing data or extreme values of key variables. We eventually retained 13,555 individuals, with 6831 in the treated group and 6724 in the control group.

### Variables

#### Dependent variable

The dependent variable in this paper is the utilization of healthcare services by migrants. Combining the questionnaires from the two waves of CMDS, we select the question “Where did you first go to see a doctor when you were last sick (injured or unwell)” to measure the migrants’ choice of healthcare services. Based on the responses of the patients who received treatment, those who received treatment at “local general/specialist hospitals” are assigned a value of 1 for visiting a high-quality medical institution, and those who received treatment at “local community hospitals”, “local private clinics”, “in their hometowns”, “other places” are assigned a value of 0. This variable is used to examine the utilization of high-quality healthcare services by migrants.

#### Independent variables

To exclude other factors that may affect the utilization of healthcare services, this paper selects independent variables from three aspects, individual characteristics, migration characteristics, and socio-economic characteristics. Specifically, individual characteristics variables included age, sex, ethnicity, marital status, education, and hukou [23–25]. Socio-economic characteristics included average monthly household income, the nature of the work unit, employment status, industry, and occupation [26]. Migration characteristics included the length of the present migration, the reason for migration [27, 28]. The definitions of independent variables are presented in Table 1 and descriptive statistics of the independent variables are presented in Table 2.

**Table 1:** Variable definition

Variables	Definition
Age	The individual’s age
Female	Male = 0, Female = 1
Ethnicity	Minority = 0, Han = 1
Marriage status	Unmarried (unmarried, divorced, widowed, cohabiting) = 0, Married (first marriage, remarriage) = 1
Education	Never went to school = 1, Primary school = 2, Junior high school = 3, High school/Secondary school = 4, Junior college = 5, Undergraduate/Postgraduate = 6
Hukou	Non-agricultural = 0, Agricultural = 1
Income	Average monthly household income (in the logarithmic form)
Nature of work unit	No unit = 1, State-owned or state-controlled enterprises = 2, Private enterprises = 3, Foreign-funded enterprises = 4
Employment status	Employee = 1, Employer = 2, Self-employment workers = 3, Others = 4
Industry	High risk industry = 1, Medium risk industry = 2, Low risk industry = 3
Occupation type	Civil servants = 1, Security, couriers, service industry workers, etc. = 2, Without fixed occupation = 3
Length of migration	Length of the migration(years)
Reason for migration	Business\Work = 1, Other (migration, caring for children, etc.) = 0

**Table 2:** Descriptive statistics of the variables

Variables	(1)	(2)	(3)
	Full Sample (*n* = 13,555)	Treated (*n* = 6831)	Control (*n* = 6724)
	Mean	Mean	Mean
Explained variable	0.290	0.323	0.263
Age	36.23	36.382	36.082
Female	0.440	0.422	0.449
Ethnicity	0.070	0.049	0.094
Marriage status	0.850	0.862	0.838
Education	3.070	3.126	3.006
Hukou	0.900	0.897	0.907
Income	8.700	8.711	8.691
Nature of work unit	2.660	2.613	2.704
Employment status	1.890	1.922	1.865
Industry	1.460	1.534	1.383
Occupation type	1.990	1.996	1.989
Length of migration	6.000	6.149	5.842
Reason for migration	0.950	0.940	0.959

Note: ***, **, * indicate statistical significance at the 1%, 5%, and 10% levels, respectively

### Econometric Strategies

Taking advantage of the quasi-natural experiment environment created by the time gap between the implementation of the immediate reimbursement in each city, we observed the impact of the immediate reimbursement policy on the utilization of healthcare services for migrants by comparing the cities that have implemented the immediate reimbursement to those that have not. Using CMDS data in 2013 and 2017, we established a DID model to investigate the impact of the trans-provincial immediate reimbursement policy on the utilization of healthcare services by migrants.

$$ {Y}_{it}={\beta }_{0}+{\beta }_{1}{X}_{it}+{\beta }_{2}{treat}_{i}*{time}_{t}+{City}_{p}+{year}_{t}+{\epsilon }_{it}$$

According to the first list of cities released by MOHRSS, we identified the inflow cities that have implemented immediate reimbursement as the treated group, $$ {treat}_{i}=1$$. The inflow cities that have not implemented immediate reimbursement as the control group, $$ {treat}_{i}=0$$. Since the policy of trans-provincial immediate reimbursement was implemented after 2016, according to the data structure of this paper, if the sample survey time is 2017, $$ {time}_{t}=1$$; if the sample survey time is 2013, $$ {time}_{t}=0 $$.The coefficient of $$ {\beta }_{2} $$of the interaction item is the key value that this paper focuses on, representing the net effect of the immediate reimbursement policy on the healthcare services utilization of the migrants in the inflow cities. $$ {X}_{it}$$ is a series of control variables, $$ {City}_{p}$$ is the city fixed effect, $$ {year}_{t}$$ represents the time fixed effect. The error term $$ {\epsilon }_{it}$$ denotes the random variability not explained by the model.

Finally, we conducted heterogeneity analysis by regressing subgroups of people with different demographic characteristics to explore the heterogeneity of policy effects. Such as industry risk, household income, education, and gender. We grouped migrants based on the health risks of the industry they work in. According to the detailed explanation of the questionnaire of CMDS, high-risk industries mainly include construction, mining, automobile manufacturing, wholesale and retail, medium-risk industries mainly include the service industry, accommodation and catering industry, and low-risk industries mainly include finance, scientific research, education, etc. Household income is divided into two groups according to the 50% quantile. In the whole sample, 54.1% of the migrants are junior high school educated, and the distribution of education is more concentrated, thus we divide the migrants into three groups according to their education level of primary school and below, junior high school, senior high school and above to carry out sub-sample analysis.

## Results

### The impact of immediate reimbursement on the utilization of healthcare services by migrants

Table 3 shows the results of the regression, and columns (1)-(4) are the results after adding control variables such as individual characteristics, migration characteristics, and socio-economic characteristics sequentially. Compared to cities without trans-provincial immediate reimbursement, the probability of the migrants in cities with trans-provincial immediate reimbursement visiting a general\specialist hospital has increased by 7%.

**Table 3:** Impact of the immediate reimbursement policy on the utilization of healthcare services by migrants

Variable name	(1)	(2)	(3)	(4)
Treat*Time	0.078^**^	0.079^**^	0.077^**^	0.075^**^
	(0.03)	(0.03)	(0.03)	(0.03)
Age		0.002^***^	0.002^***^	0.001^*^
		(0.00)	(0.00)	(0.00)
Female		0.028^***^	0.027^***^	0.027^***^
		(0.01)	(0.01)	(0.01)
Ethnicity		-0.010	-0.004	-0.002
		(0.02)	(0.02)	(0.02)
Married		-0.009	-0.036^**^	-0.038^**^
		(0.02)	(0.02)	(0.02)
Education		0.025^***^	0.019^***^	0.020^***^
		(0.00)	(0.00)	(0.00)
Hukou		-0.043^***^	-0.036^**^	-0.039^***^
		(0.01)	(0.01)	(0.01)
Income			0.043^***^	0.040^***^
			(0.01)	(0.01)
Nature of work unit				
State-owned enterprises			-0.006	-0.000
			(0.03)	(0.03)
Private enterprises			0.026^*^	0.027^*^
			(0.01)	(0.01)
Foreign-funded enterprises			0.057	0.058
			(0.03)	(0.04)
Employment status				
Employer			0.081^***^	0.073^***^
			(0.02)	(0.02)
Self-employment workers			0.044^***^	0.036^***^
			(0.01)	(0.01)
Others			0.095^**^	0.088^**^
			(0.04)	(0.04)
Industry (Benchmark group is high-risk industry)				
Medium risk industry			0.014	0.017
			(0.01)	(0.01)
Low risk industry			0.029^*^	0.029^*^
			(0.02)	(0.02)
Occupation type				
Security, couriers, service industry workers, etc.			-0.040	-0.037
			(0.02)	(0.02)
Others			-0.034	-0.034
			(0.03)	(0.03)
Length of migration (Years)				0.006^***^
				(0.00)
Reason for migration				-0.003
				(0.02)
City-fixed effect	Yes	Yes	Yes	Yes
Time-fixed effect	Yes	Yes	Yes	Yes
Observations	13,555	13,555	13,540	13,540
Adjusted R^2^	0.073	0.077	0.083	0.087

Note: ***, **, * indicate statistical significance at the 1%, 5%, and 10% levels, respectively. The numbers in parentheses are standard errors of robust SE.

### Robustness test

The robustness test in this paper is mainly performed by changing the regression model and removing the sample that may interfere.

#### Parallel trend test

Table 4 shows that there was no significant difference between the treated and control groups before the implementation of the trans-provincial immediate reimbursement policy, while there was a marked increase in the utilization of healthcare services in the treated group after the launch of the policy.

**Table 4:** Comparison of mean values of healthcare services utilization before and after the implementation of the immediate reimbursement policy

Before			After		
Treated	Control	Mean-diff	Treated	Control	Mean-diff
0.373	0.375	0.003	0.227	0.305	0.079^***^

Note: ***, **, * indicate statistical significance at the 1%, 5%, and 10% levels, respectively

In order to make the treated and control groups more comparable, this paper uses kernel matching, k-nearest-neighbor matching, and radius matching methods to match the treated group with a similar control group, respectively. As shown in columns (1)-(3) of Table 5, the estimation results of the propensity score matching difference-in-differences (PSM-DID) method show that the significance, coefficient magnitude, and the direction of the interaction term of all the equations are consistent with the baseline regression results.

**Table 5:** Impact of the immediate reimbursement policy on the utilization of healthcare services by migrants(PSM-DID)

Variable name	(1) Kernel matching	(2) K-nearest neighbor matching	(3) Radius matching
Treat*Time	0.077^**^	0.086^***^	0.75^**^
	(0.03)	(0.03)	(0.03)
Control Variables	Yes	Yes	Yes
City-fixed effect	Yes	Yes	Yes
Time-fixed effect	Yes	Yes	Yes
Observations	13,538	11,904	13,540
Adjusted R^2^	0.110	0.071	0.110

Note: ***, **, * indicate statistical significance at the 1%, 5%, and 10% levels, respectively. The numbers in parentheses are standard errors of robust SE.

#### Change model

Table 6 shows the regression results of robustness test. The results in columns (1)-(2) are the regression results of replacing the OLS model with the Probit model, and it can be found that the coefficients of interaction items are all significant, demonstrating that the regression results are robust.

**Table 6:** Robustness tests

Variable name	(1)	(2)	(3)	(4)	(5)	(6)
	Change model		Removed samples from Hainan province		Balanced panel data	
Treat*Time	0.259^***^	0.251^***^	0.074^**^	0.070^**^	0.078**	0.073**
	(0.09)	(0.09)	(0.03)	(0.03)	(0.03)	(0.03)
Control Variables		Yes		Yes		Yes
City-fixed effect	Yes	Yes	Yes	Yes	Yes	Yes
Time-fixed effect	Yes	Yes	Yes	Yes	Yes	Yes
Observations	13,256	13,243	13,160	13,146	12,944	12,929
Adjusted R^2^			0.073	0.087	0.068	0.084

Note: ***, **, * indicate statistical significance at the 1%, 5%, and 10% levels, respectively. The numbers in parentheses are standard errors of robust SE.

#### Removing potentially interfering samples

1. Removed samples from Hainan province.

Hainan Province signed agreements with other provinces for immediate reimbursement of trans-provincial payments one after another during 2009–2017, so the impact of the policy may be overestimated if migrants are flowing into Hainan Province in the sample. Columns (3)-(4) of Table 6 indicate that the coefficients of interaction term are consistent with the benchmark regression after excluding the sample from Hainan Province, proving that the results are robust.

(2) Preservation of balanced panel data.

The list of cities surveyed may change during the surveys, so not all cities included in the two waves of data are exactly the same. We retain the cities that are present in both periods of data to construct balanced panel data at the city level for the robustness test. The regression results are shown in columns (5)-(6) of Table 6, and the results are still robust.

### Heterogeneity analysis

#### Industry heterogeneity

As shown in columns (1)-(3) of Table 7, the implementation of the policy of trans-provincial immediate reimbursement significantly increases the willingness of migrants in high-risk industries to choose general/specialist hospitals in their inflow city. However, there is no significant effect on migrants in medium-risk and low-risk industries.

**Table 7:** Heterogeneity analysis: industry sub-groups and income sub-groups

Variable name	(1)	(2)	(3)	(4)	(5)
	Industry			Income	
	High-risk	Medium-risk	Low-risk	Below 50%	Above 50%
Treat*Time	0.076**	0.087	0.038	0.053	0.086^**^
	(0.03)	(0.06)	(0.07)	(0.04)	(0.04)
Control Variables	Yes	Yes	Yes	Yes	Yes
City-fixed effect	Yes	Yes	Yes	Yes	Yes
Time-fixed effect	Yes	Yes	Yes	Yes	Yes
Observations	9003	2862	1675	6078	7462
Adjusted R^2^	0.101	0.088	0.102	0.078	0.104

Note: ***, **, * indicate statistical significance at the 1%, 5%, and 10% levels, respectively. The numbers in parentheses are standard errors of robust SE.

#### Income heterogeneity

As shown in columns (4)-(5) of Table 8, the impact of household income on the utilization of healthcare services by migrants persists after the launch of immediate reimbursement. It shows that the implementation of trans-provincial immediate reimbursement mainly increases the probability of high-income groups visiting local general\specialist hospitals, while the impact on low-income migrant groups is not significant.

**Table 8:** Heterogeneity analysis: education sub-groups and gender sub-groups

Variable name	(1)	(2)	(3)	(4)
	Education			Gender
	Low-education	Medi-education	High-education	
Treat*Time	0.072	0.079**	0.088*	
	(0.05)	(0.03)	(0.05)	
Treat*Time*Gender				0.030**
				(0.01)
Control Variables	Yes	Yes	Yes	Yes
City-fixed effect	Yes	Yes	Yes	Yes
Time-fixed effect	Yes	Yes	Yes	Yes
Observations	3016	7330	3194	13,540
Adjusted R^2^	0.095	0.077	0.100	0.086

Note: ***, **, * indicate statistical significance at the 1%, 5%, and 10% levels, respectively. The numbers in parentheses are standard errors of robust SE.

#### Education heterogeneity

The regression results, shown in columns (1)-(3) of Table 8, indicate that the implementation of trans-provincial immediate reimbursement has a significant impact on migrants with junior high school education and above, while it has no significant impact on migrants with low education.

#### Gender heterogeneity

The gender variable is interacted with the core interaction term, and the regression results in column (4) of Table 8 indicate that the policy of trans-provincial immediate reimbursement has a greater impact on women than men.

## Discussion

This paper studies the impact of immediate reimbursement on the utilization of healthcare services for migrants. Similar to previous studies [29–31], the results of this paper indicate that the implementation of the immediate reimbursement policy has significantly increased the willingness of the migrants to choose more formal institutions, and improved the accessibility of high-quality healthcare resources for the migrants. The possible reason is that immediate reimbursement mitigates the liquidity constraints of the migrants in accessing healthcare services. Previous studies have found that the inconvenience of the health insurance system leads to a high proportion of migrants choosing not to receive treatment when they are sick, and preferring informal medical institutions such as individual clinics [32]. Many migrants suffer from delayed treatment due to expensive healthcare services and the inability to obtain reimbursement, and there are even cases where doctors require hospitalization, but migrants refuse treatment [33]. Thus, immediate reimbursement facilitates the utilization of healthcare services by migrants.

Further, there are differences in the impact of the policy on migrants of different genders and marital status. Migrants who are female and unmarried are shown to be more affected by the policy. Education and income also play a positive role in the policy effect on migrants. In addition, the length of migration also contributes to the policy effect, and one possible explanation is that the longer the length of migration, the more they know about the environment and policies of the inflow city.

In the heterogeneity analysis, we find some variation in policy effects. First, immediate reimbursement significantly increases the willingness of migrants in high-risk industries to use high-quality healthcare services. The reason may be that the health conditions of migrants generally vary with the industries they work in. For example, the probability of illness or injury of the migrants in the construction and mining industries is higher than that of the migrants in the finance, scientific research and education industries [34], and they are also the groups focused on the policy of trans-provincial immediate reimbursement. Therefore, implementing the immediate reimbursement policy will have a greater impact on the choice of healthcare services for this group.

Second, the immediate reimbursement policy has mainly benefited high-income migrants. In contrast, low-income migrants still have poor access to high-quality healthcare resources, which may lead to health inequality. There are variations in the liquidity constraints received by different income groups, with higher income groups tending to use more healthcare services [35]. Thus, differences in healthcare choices persist even after the implementation of the trans-provincial immediate reimbursement policy.

Additionally, the implementation of immediate reimbursement has a significant impact on migrants with higher education. This may be due to variations in the perception of policy information among migrants with different levels of education [36], which in turn leads to differences in the utilization of policies by migrants. Migrants with low levels of education have poorer perceptions of policies and thus underutilize healthcare services. This also suggests that there may be a deficiency in the policy publicity for the migrants.

Finally, we find that the policy of immediate reimbursement has a greater impact on women than men. The possible reason for this is that women are more sensitive to information about policies related to family life such as healthcare services and have a better understanding of the policy, and therefore will enjoy this policy more fully than men when they do. Another possible reason is that women use healthcare services more frequently than men, and therefore be more likely to take advantage of policy benefits.

There is a strong connection between health insurance coverage, healthcare services utilization and health. Insured patients have higher utilization rates in outpatient, inpatient, and preventive care [37], while uninsured patients are more likely to be untreated for illness due to liquidity constraints, resulting in health impairments and therefore have longer hospital stays [38, 39]. Differences in utilization of basic healthcare services among migrants due to insurance coverage led to health inequity. Inadequate utilization of basic healthcare services is an important factor affecting the health level of migrants. The low-income level and low health insurance coverage of migrants directly lead to the low accessibility of healthcare services, ultimately forming a vicious cycle of low utilization of healthcare services and poor health status [40].

The findings of this paper can be applied in three aspects. First, the government should try to narrow the differences in reimbursement rates and reimbursement categories of trans-provincial immediate reimbursement as much as possible, so that low-income migrants can also have access to adequate healthcare services. Secondly, the government should increase the publicity of the policy and simplify the operation procedures, so that migrants with low education can also fully understand the policy and make use of the healthcare services. Third, the government should expand the coverage of the policy to address inequality in service utilization by migrants.

It is worth noting that this study has certain limitations. The questionnaire’s question on the utilization of healthcare services for migrants changes slightly from year to year, leading to limitations in our choice of data years and the selection of healthcare services utilization indicators. First, we only use data from 2013 to 2017 to evaluate the effect of the policy, making it difficult to see the dynamic and long-term effects of the policy. Second, hospitalization costs are mainly reimbursed at the beginning of the policy, but due to the lack of questions in the questionnaire about whether they were hospitalized, we have to choose “Where did you first go to see a doctor when you were last sick (injured or unwell)” as the dependent variable. These issues may be better solved if more appropriate data become available in the future.

## Conclusions

This paper investigates the impact of the trans-provincial immediate reimbursement policy on the utilization of healthcare services by migrants. The results suggest that the trans-provincial immediate reimbursement significantly promotes the probability of migrants’ use of quality healthcare services. Heterogeneity analyses find that immediate reimbursement is more pronounced among high-income, better-educated, high-risk industries and female migrants. These conclusions can provide important empirical evidence for breaking up the fragmentation of health insurance and promoting the equality of basic public healthcare services.

## Data Availability

The datasets generated and/or analyzed during the current study are not publicly available due to the data confidentiality agreement, but are available from the corresponding author on reasonable request.

## Footnotes

1 Data Source: The Seventh National Population Census Bulletin (No. 7) issued by the National Bureau of Statistics.

2 Data Source: China Migrants Dynamic Survey data from 2013 to 2018.

## Abbreviations

CMDS: China Migrants Dynamic Survey
MOHRSS: The Ministry of Human Resources and Social Security of the People’s Republic of China
NHC: National Health Commission of the People’s Republic of China
DID: Difference-in-differences
